# Barriers, Facilitators, and Strategies for Developing a Culturally Informed Lifestyle Intervention for Native Hawaiian, CHamoru, and Filipino Breast Cancer Survivors: Mixed-Methods Findings from Focus Group Participants

**DOI:** 10.3390/ijerph20126075

**Published:** 2023-06-07

**Authors:** Tanisha F. Aflague, Kristi Hammond, Bernice Delos Reyes, Dareon Rios, Elaine De Leon, Rachael T. Leon Guerrero, Monica K. Esquivel

**Affiliations:** 1 College of Natural and Applied Sciences, University of Guam, Mangilao, GU 96923, USA; deleone7367@triton.uog.edu; 2 Department of Human Nutrition, Food and Animal Sciences, University of Hawai’i at Mānoa, Honolulu, HI 96822, USA; kristimh@hawaii.edu (K.H.); bernice7@hawaii.edu (B.D.R.); monicake@hawaii.edu (M.K.E.); 3 Office of Research and Sponsored Programs, University of Guam, Mangilao, GU 96923, USA; riosd@triton.uog.edu (D.R.); rachaeltlg@triton.uog.edu (R.T.L.G.)

**Keywords:** Asian Pacific Islanders, breast cancer survivors, lifestyle intervention

## Abstract

Breast cancer disproportionately impacts Native Hawaiian, CHamoru, and Filipino women. Few culturally informed interventions addressing breast cancer survivors exist and none have been developed or tested specifically for Native Hawaiian, CHamoru, and Filipino women. This study aimed to conduct focus groups with Native Hawaiian, CHamoru, and Filipino women previously diagnosed with breast cancer to inform future research in Guam and Hawai’i. Convenience sampling and grounded theory approaches were used. Focus group sessions were conducted during summer 2023 and included questions to understand the barriers, motivators, and implementation recommendations for lifestyle interventions aimed at reducing the risk for breast cancer recurrence among the target population. Data saturation was reached after a total of seven focus groups (an average of four survivors/group per site) were conducted (three in Hawai’i and four in Guam), which represented 28 breast cancer survivors. Themes from the focus groups emerged around developing support systems with other survivors, providing physical activity and nutrition intervention activities and materials in multiple formats, and incorporating activities and foods that accommodate the side effects of breast cancer treatments and are culturally relevant. The average desired intervention length was eight weeks. These findings will inform the development and feasibility testing of a culturally informed lifestyle intervention for breast cancer survivors in Guam and Hawai’i.

## 1. Introduction

Breast cancer in both Guam and Hawai’i is the second highest contributor to cancer mortality among women [1]. Breast cancer has been linked with obesity, as adipose tissue is a metabolically active endocrine organ that influences inflammatory biomarkers and tumor growth factors [2]. As such, women with obesity who are diagnosed with breast cancer experience increased relative risks for recurrence (40% to 50%) and mortality (53% to 60%) [3,4]. However, lifestyle interventions that address obesity may aid in reducing breast cancer recurrence [5,6,7].

Obesity results from a number of social determinants of health, as depicted in the social ecological model (SEM) [8,9]. The SEM demonstrates that lifestyle factors such as diet and physical activity that lead to obesity (i.e., visceral adiposity) are influenced at the individual (i.e., biologic, genetic, attitudes, beliefs), interpersonal (i.e., friends, social networks, family), organizational (i.e., churches or places of worship, work places, organizations, social institutions), community (i.e., access, design, relationships between organizations, built environment), policy (i.e., national, state, local, and organizational policies and laws) levels with sociocultural factors intersecting across these levels [10]. At the individual level, a lifestyle that includes a diet rich in fruits and vegetables and lower in ultra-processed foods and regular physical activity is protective against obesity as well as breast cancer. Adherence to these lifestyle patterns is related to upstream factors in the SEM, such as the accessibility of healthy foods, and remains poor among many populations [11,12]. The SEM reinforces the need for lifestyle interventions that address multiple levels influencing obesity risk. Evidence exists that multi-level interventions result in greater decreases in body weight, body mass index (BMI), and waist circumference compared to diet alone; however, none exist for Native Hawaiian, CHamoru, or Filipino breast cancer survivors [13]. 

Lifestyle interventions that have included cultural adaptations for Filipinos, Native Hawaiians, and/or CHamorus show promise for improved recruitment, retention, and health outcomes [14,15,16]. Community-based participatory research (CBPR) approaches and community engagement have shown to be appropriate for Native Hawaiians, other Pacific Islanders in Hawai’i, and Filipinos in developing culturally-tailored interventions and community-wide efforts [17,18,19,20]. To date, there have only been studies on these approaches applied to breast cancer screening, not lifestyle interventions [15,16].

To address the gap in lifestyle interventions for Native Hawaiian, CHamoru, or Filipino breast cancer survivors, the Traditional and New Lifestyle Interventions to Prevent Breast Cancer Recurrence (TANICA) study was initiated in 2021. TANICA was carried out in two phases. Phase I engaged community collaborators working with indigenous populations in health and research to develop an understanding of the ideal settings to promote and support behavior changes among Native Hawaiian, CHamoru, or Filipino breast cancer survivors [21]. The objectives of this research, which comprised Phase II of the TANICA study, were to: (1) identify facilitators and barriers to nutrition and physical activity lifestyle intervention components for Native Hawaiian, CHamoru, and Filipino breast cancer survivors in Guam and Hawai’i; (2) identify culturally acceptable ways of engaging women breast cancer survivors to develop a lifestyle intervention for Native Hawaiian, CHamoru, and Filipino breast cancer survivors in Guam and Hawai’i; and (3) frame facilitators and barriers across SEM levels.

## 2. Materials and Methods

### 2.1. Design

This mixed-methods study relied on focus groups to facilitate discussion around motivators, barriers, and cultural considerations for delivering lifestyle interventions in the context of the SEM in a supportive setting using a semi-structured interview guide. Pre-surveys were also conducted among focus group participants. Research activities took place at two sites, Hawai’i and Guam.

### 2.2. Participant Recruitment

A convenience sampling approach was used for recruitment. Recruitment materials were created for each research site and included information directing potential participants to an online pre-registration form to screen for eligibility. Recruitment materials were disseminated using professional networks that included, but were not limited to, community organizations, clinics, churches, and social media (i.e., Instagram and Facebook). Press releases were initiated from each research institution on Guam and Hawai’i and sent to different media outlets (e.g., radio, news TV, and web-based broadcasts). Participants who contacted their respective research site following an advertisement or announcement or who completed the pre-registration online form were encouraged to recruit other breast cancer survivors in line with snowball sampling [22]. The snowball sampling technique was adapted from public health “contact tracing” steps, in which one individual, the source, uses their social networks to recruit others, who then recruit others, and so on. This process is reminiscent of a snowball rolling down a hill. Eligible participants were later scheduled for an in-person or online focus group session.

### 2.3. Participant Eligibility

Eligible participants were: (1) Native Hawaiian and/or Filipino in Hawai’i and CHamoru and/or Filipino in Guam (participants self-reported the ethnicity with which they most identified); (2) female; (3) resident of Guam or Hawai’i; (4) 18 years of age or older; (5) proficient in English; and (6) previously diagnosed with breast cancer. English proficiency was included as the study lacked translation services. Notably, English was the primary language spoken by the target populations in this study.

### 2.4. Data Collection

Trained researchers obtained informed consent from eligible participants prior to data collection. All study materials provided and activities conducted were in English.

Surveys: All participants completed a pre-survey that obtained socio-demographic information (i.e., age and ethnicity), cultural affiliation, and quantitative assessment of preferred intervention approaches. Surveys were obtained in-person or electronically. 

Focus group protocol and procedures: Focus group discussions were guided by a semi-structured question set that consisted of key topics on cultural considerations, intervention components and strategies, delivery approaches, and place-based settings with follow-up questions specific to nutrition and physical activity. Focus group questions were designed with external reviewers, who were experts in qualitative methods and in cancer prevention research among the target ethnic groups. Interview topics included: (1) general health and cancer, (2) physical activity intervention components, (3) nutrition intervention components, and (4) intervention delivery (see Supplementary Material).

Trained moderators who lived in Guam or Hawai’i conducted focus group sessions at each site. One moderator led the discussion while the other recorded abbreviated written notes on flip chart paper (in-person) or digitally (online) and assisted with discussion probes throughout the session. All focus group sessions were audio recorded and later transcribed verbatim with personal identifiers redacted. Focus groups were capped at eight participants per session. Both in-person and online sessions were made available to accommodate participants’ preferences for participation in consideration of the COVID-19 public health emergency protocols. Focus groups were conducted until data saturation was reached. In-person sessions were held in a private room and food was provided as a cultural consideration. Online sessions were held via Zoom. 

All participants were provided with a $25 gift card at the end of each focus group session. This study was approved by both the University of Guam and University of Hawai’i Institutional Review Boards.

### 2.5. Data Analysis

Descriptive statistics were performed on the demographic and cultural affiliation data. Cultural affiliation scores are described elsewhere [21]. 

Trained researchers (two from each location) used a grounded theory approach to conduct the initial and intermediate coding of the focus group transcripts, applying constant comparative analysis. Each interview was reviewed by two independent researchers to identify themes and code transcripts. Coding based on the research aims and themes was organized by SEM level. Researchers met to determine consensus and if there were discrepancies, consensus was obtained. The most frequently mentioned themes were reviewed quantitatively. Key themes were determined if the theme was mentioned by a minimum of 50% of participants in each study location. 

## 3. Results

### 3.1. Description of Study Participants

Data saturation was reached after a total of seven focus groups were held, representing 28 breast cancer survivors (Hawai’i had one in-person and two online groups; Guam, had three in-person and one online group). Focus group participants were all female and the majority were 50 years and older (79%). In Guam, participants were either CHamoru or Filipino. In Hawai’i, the majority identified as Filipino (42%) or two or more ethnicities, which included Filipino and/or Native Hawaiian and/or another race/ethnicity (41%). Cultural affiliation mean scores were similar at each site, 8.33 for Hawai’i and 8.25 for Guam, with an overall mean score of 8.28 (Table 1). 

### 3.2. Desired Intervention Characteristics-Pre-Survey

Study participants in Hawai’i preferred physical activity interventions that were in-person (42%), group-based (50%), and in an outdoor setting (42%) (Table 2). Participants in Hawai’i preferred that the nutritional intervention activities be group-based (50%) and either online or in-person (33% for both) (Table 2). Participants in Hawai’i chose an eight-week (42%) physical activity and nutritional program held twice a week (42%) for one hour (58%), preferably during the morning hours (42%) on the weekdays (58%).

In Guam, participants favored in-person (75%), group-based (38%), physical activity intervention programs in an outdoor (56%) setting (Table 2). Most participants preferred group-based (44%) nutritional intervention activities held either in-person (38%) or online (38%) (Table 2). When asked about duration and frequency of interventions, many participants (38%) desired an eight-week-long physical activity and nutritional program held once a week (19%) with a total duration of one hour (44%). Moreover, participants in Guam found evening (44%) program sessions held mainly on the weekdays (56%) to be most favorable.

### 3.3. Barriers and Facilitators to Lifestyle Intervention

Focus group participants identified intervention strategies for diet and physical activity components that crossed multiple levels of the social ecological model at both sites (Table 3 and Table 4). Facilitators and barriers for specific lifestyle intervention components from focus group participants were identified.

#### 3.3.1. Physical Activity Barriers and Facilitators

The barriers and/or facilitators to physical activity that emerged from the focus groups in Guam and Hawai’i were: personal factors, physical limitations, accessibility and availability, healthy lifestyle, external support, and accountability (Table 3). Personal factors included gratifying and enjoyable activities, as exemplified by these participant quotes:

*“It wasn’t because of the breast cancer that prompted me to become active. I think finding something that makes me happy was the focus and it just so happened that physical activity was linked to it…maybe it’s the… serotonin and endorphins…But I wasn’t seeking a better physical life because of cancer…It just happened to come along with it”.*

—Guam Participant #6

*“I used to do a lot of running. So I enjoy running on weekends and swimming”.*

—Hawai’i Participant #10

Physical limitations appeared to be a barrier to being physically active for participants in both Guam and Hawai’i. Limitations were related to cancer treatment or existing chronic diseases. Participants in Hawai’i reported the impact of the pandemic, which limited access to spaces to be physically active as a unique barrier. Barriers unique to participants in Guam were: lack of external support related to competing responsibilities (i.e., career, familial/cultural obligations, and caring for child and/or parent), the exercise modality not being enjoyable, and safety concerns (i.e., overpopulation of stray dogs, no sidewalks/infrastructure, and crime) (Table 3). Although participants in Guam identified physical limitations as a barrier, some recognized exercises (or activities) or adaptations that facilitated being physically active, as exemplified by these quotes:

*“The thing is, I can’t walk a long distance, or I find myself limited to 15. I can push myself, maybe 30 min, because I tend to have pain in my lower back or my tailbone area. Then I feel like I have to stop, or else I might not be able to walk some more because of the pain, or I might have to sit down just to regain my strength or something like that”.*

—Guam Participant #9

*“For me, exercise was not what I thought. It was eventually after going through the chemo treatment and the radiation, my body was just so tired, it took me about two to three years to finally get my stamina going back again”.*

—Hawai’i Participant #4

Facilitators common to both sites were: exercises (or activities) that were enjoyable and/or part of a healthy lifestyle routine, and support/accountability (Table 3). Participants in Hawai’i were the only participants to identify lab work and other clinical measures as facilitators of physical activity interventions, specifically noting that these could support accountability. Guam participants shared a willingness to participate in lab and biospecimen collection. In Guam, participants acknowledged that there were few specific areas/spaces that were accessible for safe physical activity based on proximity to neighborhood or village recreation centers.

#### 3.3.2. Nutrition Barriers and Facilitators

The barriers and/or facilitators to healthy diet behaviors identified by the focus groups at both sites were: accessibility and availability, external factors, and lack of information about nutrition during diagnosis/treatment (Table 4).

Nutrition information was both a barrier and a facilitator. A common barrier at both sites was a lack of nutrition information. Meanwhile, participants in Hawai’i stated nutrition education (e.g., cooking, sampling, menu planning), assistance, and accessibility (prepared for participants) as facilitators of a healthy diet and lifestyle intervention program. In Guam, participants noted specific nutrition education topics such as nutrition for cancer and local (modified) recipes. The need for nutrition information is captured in these quotes:

*“the types of foods that prevent cancer, because I do know that there are those types…for cancer, preventative and then I guess after cancer... I know that during chemo, there are certain things that people don’t want to eat because they have that metallic taste, so things to combat that...Even maybe recipes in some type of booklet or a pamphlet...I know I never got that and I always wanted that”.*

—Guam Participant #1

*“The problem I have with that kind of thing is with my cancer is a lot of the things that I could eat before are [inaudible]. The essential oils, I cannot have that. I cannot have sesame seeds anymore. …For me, I’m looking for is what things can I eat? What things do I need to avoid I really had to cut out a lot of things…”*

—Hawai’i Participant #7

Accessibility and availability were mentioned both as barriers and facilitators. In Guam and Hawai’i, high food costs were a barrier to the accessibility and availability of healthy food. However, the availability of local (seasonal and healthy) ingredients was viewed as a facilitator. External factors were both barriers and facilitators to healthy eating in Guam. Within the social structure, social obligations related to attending gatherings and eating were barriers, yet tight social networks were a support system that facilitated healthy eating. 

#### 3.3.3. Intervention Strategies across Social Ecological Model (SEM) Levels

Intervention strategies at the individual level were underscored with personalization that would meet the individual’s needs, such as addressing health issues or work/family schedule and/or developing and monitoring personal goals. Participants expressed support and accountability factors that reflected *interpersonal* strategies to promote physical activity as well as a healthy diet (Table 3). Multiple intervention formats as a vehicle for support at the *individual* and *interpersonal* levels are exemplified by the following quotes:

*“And so having avenues like this in-person or online, I think is very helpful for survivors, because then it creates that type of community that we’re not alone…we do understand the strength it takes to continue to survive and stay hopeful”.*

—Hawai’i Participant #1

*“And if there’s going to be a recipe for those locally available ingredients, I wish that it is available online so that for those who don’t have time-- I mean, it is nice to make that resource very convenient for everyone”.*

—Guam Participant #4

At the organizational level, utilizing existing community programs for both physical activity and nutrition or food education was identified. In Guam, existing programs were the source of trusted information, such as a credentialed or trained health professional, or where other breast cancer survivors could share their lived experiences. Community-level strategies only emerged for the physical activity intervention components among participants in Guam, which are outlined in Table 3. Safety and accessibility emerged as barriers to physical activity in Guam, revealing a policy-level intervention strategy to be addressed. Both sites identified high food costs as a barrier to healthy eating, which is at the policy level of the SEM.

#### 3.3.4. Cultural Considerations

Sociocultural intervention components intersecting all SEM levels addressed shared cultural values among all ethnic groups and social norms at each research site. Family-centered considerations appeared in both nutrition and physical activity components at both sites. In Hawai’i, sharing stories or “talk-story” was a component of nutrition and physical activity. In Guam, a participant suggested cultural shifts to community events, for example:

*We can shift by flipping the fiesta table. Maybe that’s a project that we can do or something, have the vegetables first”.*

—Guam Participant #6

*“We need to normalize walking [referring to active transport]”.*

—Guam Participant #6

One participant in Hawai’i identified the strategy to recruit only Native Hawaiian and Filipino women as attractive. This quote captures the consideration to include representative cultures and ethnic groups of the geographic site:

*“And I think what attracted me to this group, I think I did see it in the paper was the Native Hawaiian and Filipino aspect of it. If it was a bigger, just are you a breast cancer survivor? I’m not so sure I would’ve picked up the... emailed or however I made that first connection. It was that specific cultural thing that drew me in”.*

—Hawai’i Participant #5

## 4. Discussion

This was the first study to use focus groups to capture barriers and facilitators to eating healthy and being physically active among CHamoru and Filipino breast cancer survivors in Guam and Native Hawaiian and Filipino breast cancer survivors in Hawai’i for the purpose of developing a lifestyle intervention. The focus groups consisted of mixed ethnic groups (i.e., Native Hawaiian and Filipino or CHamoru and Filipino) at both sites, yet many of the facilitators and barriers identified were similar and participants agreed with others’ contributions, revealing place-based motivators and challenges unique to each site. The findings also supported having interventions for mixed groups grounded in the shared cultural values and practices and the island environment and resources.

Participants in Guam and Hawai’i preferred an eight-week intervention, which aligned with a number of existing health interventions that target lifestyle changes for Native Hawaiian, Pacific Islander, or Filipino groups. Some examples included the Wahine Heart Wellness Program, which is a cardiovascular disease education program for Asian, Native Hawaiian, and Pacific Islander women [23], and another physical activity intervention trial for Filipino women involving Zumba [24]. Differences in intervention meeting frequency were noted among participants in Guam and Hawai’i. In Guam, TANICA participants preferred 60 min, once a week. Rock et al. [25] employed a similar weekly one hour group session. However, their intervention tapered to every other week for two months, and monthly for six months over a 24-month period [25]. The Kā-HOLO project by Kaholokula et al. [26] involved two 60 min hula sessions each week, similar to the request by the TANICA Hawai’i participants. TANICA study participants at both sites also requested outdoor physical activities, which was similar to published studies. For example, one study included an intervention that was held at outdoor parks [27]. 

Group-based, in-person physical activities were requested by participants in Guam and Hawai’i, similar to other interventions such as the “Siglang Buhay” intervention [28], Filipinos Fit & Trim [15,29], the eight-week Zumba intervention [24], and the Kā-HOLO project [26]. Although in-person physical and nutrition interventions were favored by the TANICA participants in Hawai’i (42%) and Guam (75%), the use of either online or in-person support was the second favored option in Hawai’i (33%) and Guam (19%). Previous interventions have been successful using this mix of components [15,29]. One example, the Filipinos Fit & Trim study [15,29], included in-person weight checks and personal coaching as well as in-person meetings, digital applications, and social media for activity and nutrition tracking and social support. Playdon et al. [30] also utilized both in-person and telephone weight loss counseling in one-on-one and group settings, similar to the preference of the TANICA participants. 

Lastly, TANICA participants recognized the need for nutrition and physical activity interventions that address multiple facets of themselves, such their families, communities, and cultures. In Guam, Filipino participants emphasized their faith as a facet of themselves to be recognized. This was supported by Playdon et al. [30], who also found more success using a multi-component (diet, physical activity, and behavior modification) intervention rather than single-component interventions.

The “talk story” approach has been documented in qualitative research as a culturally acceptable strategy used with Native Hawaiian and other Pacific Islanders and in this study, it was identified as a component to include in a lifestyle intervention through social support [31]. This alignment was expected as Pacific cultures rely on oral history using storytelling to pass on traditions, family lineages, and recipes [32,33,34,35].

The timing of this study revealed an unexpected finding, which was the impact of the COVID-19 pandemic on establishing and maintaining healthy behaviors for breast cancer survivors. At both study sites, women reported that they started or learned a new physical activity and experienced challenges to being active due to indoor gyms or public spaces being inaccessible during the pandemic. For many, new or sustained physical activity was outdoors and related to indoor restrictions, which may have made women more likely to prefer outdoor activities during the focus group sessions. Another impact of the pandemic was the increased willingness to participate in virtual learning and openness to receive digital or online resources (e.g., e-newsletters, YouTube videos, apps), which was related to the frequent use of virtual meeting platforms and digital public service announcements [36,37,38,39,40,41]. Women shared that their comfort level and proficiency of using online or digital resources improved during the pandemic due to the physical restrictions that forced them to use and/or learn them.

This novel study investigated facilitators and barriers to lifestyle interventions across the SEM. This information provides unique insight into the women’s lived experiences and key levels of the SEM to address. Interestingly, all levels of the SEM were not represented in the facilitators and barriers mentioned by participants, which differed between the two sites and by component type (physical activity or nutrition). For example, in Guam, barriers and/or facilitators were identified at all levels, except the organizational level for physical activity and the community level for the nutrition component. In Hawai’i, barriers and/or facilitators were mentioned for four of six SEM levels for the physical activity (individual, interpersonal, community, and sociocultural) and nutrition (individual, organizational, policy, and sociocultural) components. There were parallel findings between research sites in that participants did not identify barriers and/or facilitators at the organizational and community levels for physical activity and nutrition components, respectively. This demonstrated possible similarities between sites. Unsurprisingly, *sociocultural*-level barriers and facilitators were identified at both sites and across components. These findings underscored the unique needs of each population. Together, this information reinforces the need for place-based and adapted interventions to best serve this population. 

A strength of this qualitative study was that it addressed and met the 21 criteria of the Standards for Reporting Qualitative Research Guidelines [42]. Limitations of this research included the mixed in-person and virtual participation methods, relatively small focus groups (three to five versus six to ten), and the inclusion of only English-speaking participants due to the researchers and research staff being proficient in English. However, English is one of the official languages in both locations, while Hawaiian and CHamoru are the other languages in Hawai’i and Guam, respectively. In addition, the groups were not moderated by a breast cancer survivor; although the moderators were familiar with the populations and place, they were not survivors. Because the focus group sessions were conducted during the COVID-19 pandemic, the results may not represent the women’s real preference for physical activity and nutrition intervention activities post-pandemic. Lastly, the findings may only be relevant to the two geographic locations and may not apply to the target population living in other places. In Hawai’i, participants represented specific locales, Oʻahu, Maui, and Hawai’i Island, which are three of the eight Hawaiian islands in the state. 

## 5. Conclusions

Developing support systems with other survivors, providing physical activity and nutrition intervention activities and materials in multiple formats, and incorporating activities and foods that accommodate the side effects of breast cancer treatments and are culturally relevant were key themes. The average desired intervention length was eight weeks. The findings of the study were supported by a number of published interventions that targeted similar ethnic groups but with a focus on different health conditions (i.e., cardiovascular disease, diabetes). These findings will be used immediately to inform the development and feasibility testing of a lifestyle intervention aimed at reducing obesity and improving the diet and physical activity of Native Hawaiian, CHamoru, and Filipino breast cancer survivors. These findings may also be of interest to others working to develop interventions within the Native Hawaiian, CHamoru, and Filipino communities in Hawai’i and Guam. 

## Figures and Tables

**Table 1:** Characteristics of female breast cancer survivors who participated in focus groups in Guam and Hawai’i.

	Guam *n* = 16	Hawai’i *n* = 12	Total *n* = 28
**Age Group**	***n* (%)**		
<29 years	0 (0)	1 (8)	1 (3)
30–39 years	1 (6)	0 (0)	1 (3)
40–49 years	3 (19)	1 (8)	4 (15)
50 years and older	12 (75)	10 (84)	22 (79)
**Race/Ethnicity**	***n* (%)**		
CHamoru	8 (50)	0 (0)	8 (29)
Filipino	7 (44)	5 (42)	12 (43)
Native Hawaiian	0 (0)	2 (17)	2 (7)
Filipino & Asian	0 (0)	1 (8)	1 (3.3)
Native Hawaiian & Filipino	0 (0)	1 (8)	1 (3.3)
Native Hawaiian and/or Filipino, & other ethnicity ^1^	0 (0)	3 (25)	3 (11)
Not specified	1 (6)	0 (0)	1 (3.3)
**Cultural Affiliation**	**Mean ± SD**		
Mean Score ± SD ^a^	8.25 ± 3.39	8.33 ± 3.55	8.28 ± 3.39

^1^ Combination of three or more ethnicities, including Native Hawaiian and/or Filipino. ^a^ SD = standard deviation.

**Table 2:** Desired characteristics of a lifestyle intervention for Native Hawaiian, Filipino, and CHamoru breast cancer survivors in Guam and Hawai’i.

	Hawai’i *n* (%)		Guam *n* (%)		Total *n* (%)	
	Physical Activity	Nutrition	Physical Activity	Nutrition	Physical Activity	Nutrition
**Intervention Type**						
Group only	6 (50)	6 (50)	6 (38)	7 (44)	12 (43)	13 (46)
Individual only	2 (17)	2 (17)	5 (31)	5 (31)	7 (25)	7 (25)
Either	3 (25)	3 (25)	5 (31)	5 (31)	7 (25)	7 (25)
No Response	1 (8)	1 (8)	0 (0)	0 (0)	1 (4)	1 (4)
**Delivery Mode**						
In-person only	5 (42)	4 (33)	12 (75)	6 (38)	17 (61)	10 (36)
Online only	2 (17)	4 (33)	1 (6)	6 (38)	3 (11)	10 (36)
Either	4 (33)	3 (25)	3 (19)	4 (25)	7 (25)	7 (25)
No Response	1 (8)	1 (8)	0 (0)	0 (0)	1 (4)	1 (4)
**Location**						
Outdoor only	5 (42)	--	9 (56)	--	14 (50)	--
Indoor only	2 (17)	--	2 (13)	--	4 (14)	--
Either	4 (33)	--	5 (31)	--	9 (32)	--
No response	1 (8)	--	0 (0)	--	1 (4)	--

**Table 3:** Intervention strategies informed by facilitators and barriers to physical activity components of a lifestyle intervention identified by breast cancer survivors in Guam and Hawai’i, organized by social ecological model (SEM) level.

SEM Level	Intervention Components for Physical Activity Components	Perspectives and Illustrative Quotes
Individual	Guam Personal goal setting, achievements, and incentives Hawai’i Activity performed individually because of lifestyle and career and at most convenient place for individual (e.g., home or at gym) Being kept accountable with apps and devices (i.e., Fitbit)	“What’s my goal? Maybe it was never to change my diet. So that’s why it’s not important for me. But if we all had a common goal. Like, “Look guys. Little by little, we’re changing whatever”. Then how we get there is up to us”. —Guam Participant #6 “…have some kind of measuring to where we would log it to you or on a log sheet or whatever we do in terms of an activity a day and for the week. What I see is that accountability for myself to make sure that I do this thing, because sometimes they’re like, ‘Okay,’ then we eat our sweets. We don’t know if it is the sweets or not, but to be accountable and say, ‘Hey, you know what? I want to be a part of this? Do I want to go walking 30 min a day so I can log it in.’ To me, that’s accountability and I would love to be a part of that”. —Hawai’i Participant #6
Interpersonal	Guam Exercise with a support system (e.g., family, partner, or other individuals for accountability) Hawai’i Being in a supportive group	“Motivation. I need consistency and motivation...A core group of people who I can do it with. They depend on me and then I depend on them... So if you have someone that [is] relying on you to meet them there and then you feel obligated to be there, and then it works both ways”. —Guam Participant #1 “But in the group exercise that we do, it’s good because you have other people that are exercising with you, so it motivates you to come to class, because I know that our group has gotten pretty close. And so, if somebody is not going to be there, we have to answer to why that is. That part. That part of it is good”. —Hawai’i Participant #12
Organizational	Hawai’i Indoor (preferred) location with childcare Utilizing or partnering with existing community resources/programs for nutrition education or counseling, physical activity, mental health, or stress management	“We would go there (community center) like 9:30 in morning, twice a week or two times a week. But that all stopped during the pandemic. So that community helped me a lot. Socializing with people”. —Hawai’i Participant #8
Community	Guam Family-friendly environment (e.g., park, hotel, hotel pool, beach)	“walking by the beach or at the complex…a cemented path… even here at Ypao…or at the complex…up in Dededo, we go five rounds and then when we’re so late in the morning, we just walk in the neighborhood because we have a very quiet neighborhood, no dogs… no everything. So it’s, really, not much cars”. —Guam Participant #5
Policy	Guam Safe environment (e.g., address stray dogs and crime at physical activity spaces)	“I used to walk in Asan, but then we had those reports of people breaking into cars”. —Guam Participant #3 “And it goes back to the sidewalks. Our physical setup of the island or the city isn’t conducive to walking...so even for our younger kids to walk to the store, it’s right there, but it’s not safe because the road and the puddles so if we’re not setting our island up to live that healthy lifestyle…” —Guam Participant #6
Sociocultural (across all levels)	Guam Family-centered: family can be a support system to perform physical activity together Active transport (e.g., walking to commute) not normalized Hawai’i Family-centered: family can be a support system to perform physical activity together Group activities with similar cultural backgrounds Sharing and exchanging stories with other survivors	“Obligatory events. If you have something that’s planned by the family, you better go to that and forget your exercising. And I do that, I honor that because my family does come first”. —Guam Participant #7 “And so having avenues like this in-person or online, I think is very helpful for survivors, because then it creates that type of community that we’re not alone. We’re all walking through this together. And we might not have had the same treatments or the same different things that happen to us, but we do understand the strength it takes to continue to survive and stay hopeful”. —Hawai’i Participant #1

**Table 4:** Intervention strategies informed by facilitators and barriers to nutrition components of a lifestyle intervention identified by breast cancer survivors in Guam and Hawai’i, organized by social ecological model (SEM) level.

SEM Level	Intervention Strategies for Nutrition Components	Perspectives and Illustrative Quotes
Individual	Guam Prepared foods for busy individuals Consider individual health issues (i.e., diabetes, hypertension, high cholesterol) Online resources available with in-person events Hawai’i Online or in-person in location that is culturally comfortable (e.g., individual in own home or group at hotel or *lo’i (taro patch in Hawaiian)*)	“a nutrition class focusing on locally available foods....because if we say, oh, you need to eat this and it’s only available in the mainland or it costs $10 a pound... you can’t motivate people to eat that all if it’s going to cost that much. So locally available, hopefully not too costly”. —Guam Participant #3 “And they [University of Hawaiʻi] have the loʻi right?... But I think that would be a good place”. —Hawai’i Participant #10
Interpersonal	Guam Adapt local recipes, such as brown red rice, tofu *kelaguen (animal protein in citrus marinade in CHamoru)* Culturally appropriate nutrition content	“I so agree with the local foods, I think affordability and availability and freshness of it is very important to consider. And if there’s going to be a recipe for those locally available ingredients, I wish that it is available online so that for those who don’t have time--I mean, it is nice to make that resource very convenient for everyone”. —Guam Participant #4 “And even if you pick two things, things that you can actually grow here and then different ways that you can prepare it…finding alternate recipes [referring to cultural recipes]” —Guam Participant #16
Organizational	Guam Utilizing or partnering with existing community resources/programs (trusted source) for nutrition education, cooking classes, recipes, and gardening Topics: nutrition specific for cancer survivors and recipes that include healthy alternatives and local foods Pre-cooked meal options Hawai’i Healthier (local) alternatives and affordable menu/meal plans when eating out Pre-cooked meal options Nutrition education	“If there is a cooking class or someone’s house, if they’re comfortable or even in a restaurant… If there’s certain restaurants that maybe some of the survivors or patients like, and then they can get tips from those restaurants, they can sponsor the thing”. —Guam Participant #9 “So what made it easy for you, because we all work. You don’t have time, and I don’t have time, to cook. I really don’t. I work until late, that’s bad for me. They give you the food for you. [in reference to prepared foods in program]” —Guam Participant #13 “Yeah. I like that idea of integrating the food that we have here on the islands because it’s hard when you see all these different diets and you’re like, ‘Yeah, that’s cheap on the mainland, but here when we go to [wholesale store], it’s expensive. Everything is so expensive.’ So being able to integrate what we do have in terms of even the food that’s available here”. —Hawai’i Participant #1
Community	No data	No data
Policy	Guam Affordability and accessibility of healthy food Hawai’i High food cost	“I so agree with the local foods, I think affordability and availability and freshness of it is very important to consider”. —Guam Participant #4 “So even being aware of how expensive it is here and the food that we have that is available here all year round, not the ones that are shipped or the ones that go up and down with prices, but what is available, integrating that with menu and food preparation and all that, I think would help the local community here as well”. —Hawai’i Participant #1
Sociocultural (across all levels)	Guam Involve family in nutrition education due to food-centric culture Include *fiestas (celebration gatherings in CHamoru)* and *fiesta* food in education (e.g., healthy modifications to traditional foods and *fiesta* tables) Hawai’i Family-centered: family can be a support system for eating healthy together Sharing and exchanging stories with other survivors	“You remind me though, when you’re in Guam, you have to practically attend everything that happens, right?… So that is a stress factor, too, just being in here. Although family support is really good, it gets too much. So kind of learning to say, ‘No, I’m not coming’”. —Guam Participant #14 “For me, I would just have to do what I really... sometimes, no offense, I don’t want to do, but I got to do it if I want to live longer. If I want to be here to see the grandsons graduate”. —Hawai’i Participant #11

## Data Availability

Not applicable.

## Supplementary Materials

The following supporting information can be downloaded at: https://www.mdpi.com/article/10.3390/ijerph20126075/s1, File S1: TANICA: Focus Group Questions.
