# Supplementary material for: Barriers, Facilitators, and Strategies for Developing a Culturally Informed Lifestyle Intervention for Native Hawaiian, CHamoru, and Filipino Breast Cancer Survivors: Mixed-Methods Findings from Focus Group Participants
Source: Int J Environ Res Public Health. 2023 Jun 7;20(12):6075. doi: 10.3390/ijerph20126075 (PMC10298141; doi:10.3390/ijerph20126075)
Supplement: Supplementary file 1 [file ijerph-20-06075-s001.zip › ijerph-2332431-supplementary.pdf]

File S1

TANICA: Focus Group Questions

Introduction of Participants:

Moderator: *Could we begin by each of you telling us a little something about yourself [like your favorite leisure activity]? Let's start with you [address one participant].*

Note: Probes will only be used after asking questions and not getting a response or to redirect focus on a second round of responses.

Section 1 – General Health and Cancer:

(10 MINS.)

1. What first comes to your mind when you hear "diagnosed with cancer"?

2. What does health or being healthy mean to you? What do you think of? How does it feel?

Moderator: *Throughout today's talk story session recall experiences from your journey and how you adapted or coped. Also, think of what you would of done differently during your journey.*

TRANSITION INTO PHYSICAL ACTIVITY INTERVENTION DISCUSSION

Moderator: *Evidence suggests that lifestyle interventions that promote a healthy body weight can reduce breast cancer recurrence. Research shows independent lifestyle factors that promote survival are physical activity and a diet high in fiber and lower in saturated fat. Yet these studies have taken place outside of Guam and Hawai'i and include few, if any, Asians and Pacific Islanders.*

To develop a lifestyle intervention that best fits Native Hawaiian/CHamoru and Filipino breast cancer survivors. Let's talk about physical activity.

Section 2 – Physical Activity Interventions

(30 MINS.)

1. What kind of physical activities do you most enjoy? and Why?
   Probe: What would make physical activity or exercise more fun?

2. What kind of exercise activities are you consistent with (e.g., 3 days per week)? and Why?
Probe: If exercise is not currently part of your lifestyle, what activities would appeal to you or what would encourage you to start?

3. Where would you like to participate in regular physical activity?
Probe: What community organization should be involved in delivering a physical activity program?
Examples of community organizations: Zumba, Boot Camp, Beach Swimming, Sports Clubs (e.g., paddling, volleyball, etc.), Gyms
Probe: Is there anyone you would recommend from the community organization identified ?

4. What do Filipino/CHamoru/Native Hawaiian breast cancer survivors need to be successful in a physical activity program?
Note: "Successful" is to complete the program as designed AND continue activities after the program is over.
Probe: What aspects of Filipino/CHamoru/Native Hawaiian culture can be used to encourage and/or support physical activity? How?

5. What are the cultural considerations to participating in physical activity?
Probe: What are the cultural considerations at any of the settings mentioned in Question 3?

TRANSITION INTO POTENTIAL NUTRITION INTERVENTIONS DISCUSSION

Moderator: *We've talked some about physical activity interventions Now let's talk a little more about nutrition interventions*

Section 3 – Nutrition Interventions
(30 MINS.)

1. What kind of food and nutrition topics and/or activities would be most helpful in a program?
Probe: What gets in the way of making healthy food and beverage choices?

2. What do Filipino/CHamoru/Native Hawaiian breast cancer survivors need to be successful in a nutrition education/food activity?
Note: "Successful" is to complete the program as designed AND continue activities after the program is over.

Probe: What do Filipino/CHamoru/Native Hawaiian breast cancer survivors need to make long-lasting healthy food and beverage choices?

3. What are the cultural considerations to participating in nutrition education/food activity?
Probe: What kind of foods would you want to eat/prepare during a diet/nutrition intervention class? Where or how would you get these foods?

4. Where would you like to participate in nutrition education/food activity?
Probe: What community organization should be involved in delivering a nutrition education/food activity program?

5. Who should provide the nutrition education/food activity?

TRANSITION INTO POTENTIAL INTERVENTION DELIVERY DISCUSSION

Moderator: *We've talked some about physical activity and nutrition interventions Now let's talk a little more about potential ideas for setting up the program.*

Section 4 – Intervention Delivery
(30 INS.)

1. How long do you think an intervention should be? Some models are weekly for the first 2 months, then visits get more spread out over time, how would that work for you?

2. Describe your support system as a breast cancer survivor?
Probe: How can your support system be engaged in a lifestyle program?

3. [For Moderator: ask all questions together, then go around] How would you use digital technology such as apps (nutrition and activity trackers), wearable technology (Fitbit or other wearable sensors) in supporting healthy lifestyle changes?

4. What would be the best way to communicate with breast cancer survivors participating in a lifestyle intervention (i.e., social media group, phone calls, emails, regular group meetings, through an app)?

5. What do you think are important ways that we could measure the impact of the intervention on your health? Note: complete round 1 of responses before asking probes.

Probes: Would you be comfortable with any of the following: measuring weight on scale, measuring waist circumference, measuring body fat composition in machine, labwork (stool, saliva, blood, urine)? What would make you more comfortable with these types of health measurements?

6. What would motivate you to participate in a lifestyle intervention?
   Examples: prizes, competition, teamwork, support group, etc.
   Probe: If prizes (or incentives mentioned) given in response, ask what type?

7. Should the lifestyle intervention include a stress management or mental health component? *If so, what are sources of stress? What tools or strategies do you use or know of to manage your stress?*

Closing

(10 MINS.)

Moderator: *We want to thank you for taking time to talk with us today. We have brought some healthy snacks for you to enjoy. We will be glad to stick around for a while to answer any other questions you may have.*
